# Maple syrup urine disease due to a paracentric inversion of chr 19 that disrupts *BCKDHA* : A case report

**DOI:** 10.1002/jmd2.12333

**Published:** 2022-09-20

**Authors:** Katsuyuki Yokoi, Yoko Nakajima, Yuta Sudo, Tasuku Mariya, Rie Kawamura, Makiko Tsutsumi, Hidehito Inagaki, Tetsushi Yoshikawa, Tetsuya Ito, Hiroki Kurahashi

**Affiliations:** ^1^ Department of Pediatrics Fujita Health University School of Medicine Toyoake Japan; ^2^ Division of Molecular Genetics Institute for Comprehensive Medical Science, Fujita Health University Toyoake Japan; ^3^ Department of Obstetrics and Gynecology Sapporo Medical University School of Medicine Sapporo Japan

**Keywords:** *BCKDHA*, FISH, maple syrup urine disease, paracentric inversion of Chromosome 19, whole‐exome sequencing

## Abstract

Maple syrup urine disease (MSUD) is a rare autosomal recessive inherited disorder of branched‐chain amino acid metabolism caused by mutations in *BCKDHA*, *BCKDHB*, and *DBT* that encode the E1α, E1β, and E2 subunits of the branched‐chain α‐ketoacid dehydrogenase (BCKD) complex. Various MSUD‐causing variants have been described; however, no structural rearrangements in *BCKDHA* have been reported to cause the classic MSUD phenotype. Here, we describe the classic patient with MSUD with compound heterozygous pathogenic variants in *BCKDHA*: a missense variant (NM_000709.3:c.757G > A, NP_000700.1:p.Ala253Thr) and a paracentric inversion disrupting Intron 1 of *BCKDHA*, which was identified by whole‐genome sequencing and validated by fluorescence in situ hybridization. Using the sequence information of the breakpoint junction, we gained mechanistic insight into the development of this structural rearrangement. Furthermore, the establishment of junction‐specific polymerase chain reaction could facilitate identification of the variant in case carrier or future prenatal/preimplantation tests are necessary.

**Synopsis**

We report the classic form of maple syrup urine disease with paracentric inversion of Chromosome 19, which disrupts *BCKDHA*.

## 1 INTRODUCTION

Maple syrup urine disease (MSUD, OMIM 248600) is a rare autosomal recessively inherited inborn metabolism error caused by deficiency in the branched‐chain alpha‐keto acid dehydrogenase (BCKD) complex.^1^ The deficiency of BCKD causes the corresponding branched‐chain keto acids (BCKAs) formed by branched‐chain amino acid (BCAA) transaminase to be unable to oxidize dicarboxylic acid, resulting in the accumulation of BCAAs (including leucine, isoleucine, and valine), and BCKAs.^2^ BCKD comprises three catalytic components: a branched‐chain α‐keto acid decarboxylase (E1) formed by two E1α and two E1β subunits, a dihydrolipoyl transacylase (E2), and a dihydrolipoamide dehydrogenase (E3), encoded by *BCKDHA*, *BCKDHB*, *DBT*, and *DLD*, respectively.^3^, ^4^ Based on clinical presentation onset age and residual BCKD complex activity, MSUD can be divided into four forms: classic, intermediate, intermittent, and thiamine responsive.^5^, ^6^ Patients with the classic phenotype of MSUD have <3% residual BCKD complex activity and a clinical onset typically in the first weeks of life.^7^ *BCKDHA* consists of nine exons and is located on Chromosome 19q13.2.^8^ Although many MSUD‐causing variants have been identified in *BCKDHA*,^5^, ^9^ no structural rearrangements have been reported as causative of MSUD. Herein, we describe the classic form of MSUD with paracentric inversion of Chromosome 19, which disrupts *BCKDHA*, and present evidence derived from molecular tests. Furthermore, we showed that whole‐genome sequencing is a powerful tool for diagnosing genetic diseases because of its potential to detect pathogenic variants in coding and noncoding regions, copy number variations, and balanced structural rearrangements.

### 1.1 Case

The female patient was the third child of nonconsanguineous parents from Japan, with a birth weight of 2366 g. At birth, she was found to have mild hypoglycemia and was temporarily administered a glucose infusion till 5 days of age. She tested positive for MSUD in a newborn screening (NBS) performed at 5 days of age. The concentration of BCAAs in dried blood spot at NBS were markedly elevated with valine 776.7 nmol/ml (normal range 46.16–231.7), leucine 1646.7 nmol/ml (normal range 57.16–246.95), and isoleucine 526 nmol/ml (normal range 36.2–112.5). At 8 days of age, she was hospitalized and noted to have metabolic acidosis (pH 7.327, pCO2 20.7 mmHg, HCO_3_^−^ 10.9 mmol/l, BE −10.9 mmol/l) and hypoglycemia (glucose 1.8 mmol/l). She was immediately treated with glucose infusion and diet therapy, with restricted BCAA intake. The plasma levels of BCAA gradually decreased, and consequently, metabolic acidosis improved. Brain magnetic resonance imaging performed at 1 month of age showed no abnormalities. The plasma concentrations of BCAA in the patient were strictly monitored after discharge, and the leucine concentration was maintained at a safe level for MSUD treatment (150–400 nmol/ml). However, she had several metabolic decompensations triggered by infections and was admitted to the intensive care unit three times because of severe metabolic acidosis with impaired consciousness. Currently, she is 10 years of age. Her height and body weight were 141.6 cm (z‐score, 0.0) and 39.9 kg (z‐score, 0.5), respectively. The intellectual quotient (IQ) score was obtained using the Tanaka‐Binet Intelligence Scale (Japanese version of the Stanford‐Binet Intelligence Scale). The patient's IQ score was 44. The patient was clinically and biochemically diagnosed with MSUD, and genetic testing was performed to confirm the diagnosis.

### 1.2 Genetic testing

**FIGURE 1:**
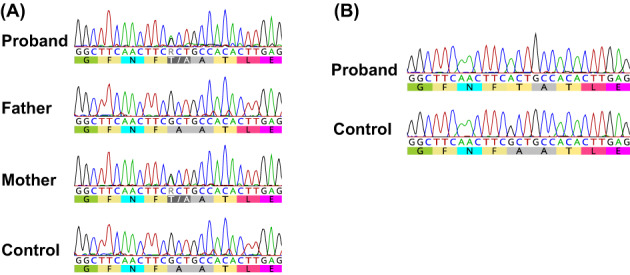
*BCKDHA* Sanger sequencing results. (A) Using genomic DNA as a template, the region containing c.757G > A (p.Ala253Thr) was analyzed by Sanger sequencing. Nucleotide and amino acid sequence data are shown at the bottom of the sequence data. (B) Using cDNA as a template, the region containing c.757G > A (p.Ala253Thr) was analyzed by Sanger sequencing.

**FIGURE 2:**
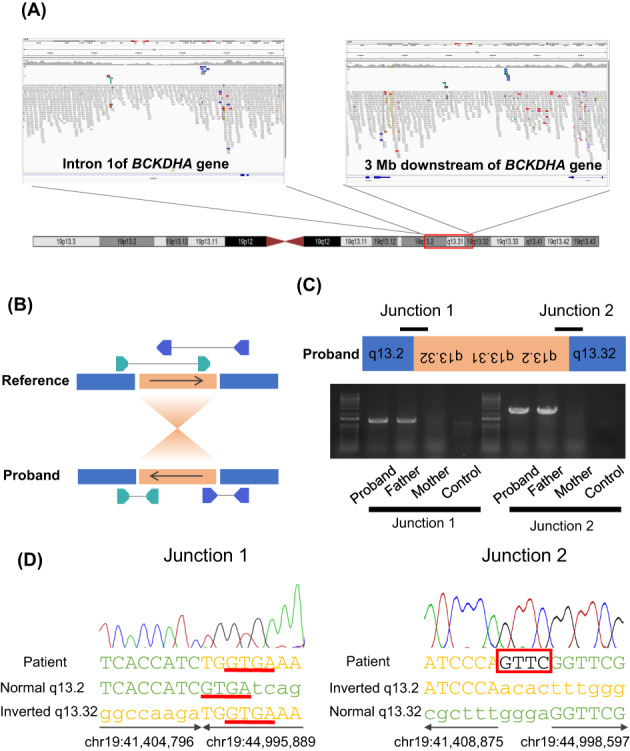
Results of other genetic tests. (A) integrative genomic viewer view of the paracentric inversion region. The discordant reads are labeled with yellow, green, and blue. (B) Structural rearrangements are determined from the orientation and distance of discordant reads. In the inversion, both reads are oriented in the tandem direction. (C) Predicted structure of the junction and inversion‐specific polymerase chain reaction (PCR). The PCR primer pair successfully amplified the junction product only from DNA from the proband and her father. (D) Sanger sequencing results for the PCR products including the junctions. The normal sequences are aligned in green, and inverted sequences are aligned in yellow. Sequences that are lost in the patient are indicated by lowercase letters. Underlined nucleotides indicate microhomology at Junction 1. Red box denotes a microinsertion at Junction 2.

**FIGURE 3:**
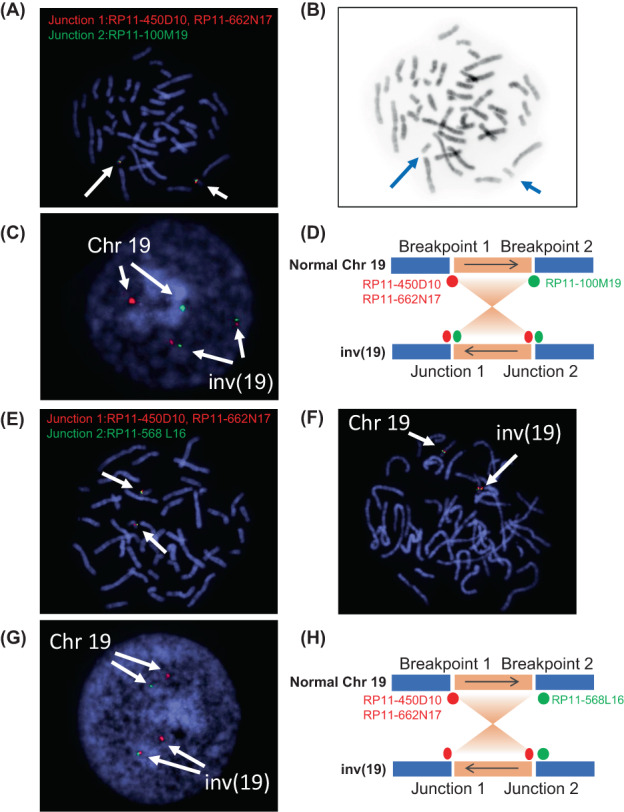
Fluorescence in situ hybridization (FISH) analysis results. (A–D) FISH analysis of peripheral blood cells using probes of breakpoints 1 (RP11‐450D10, RP11‐662 N17; Red) and 2 (RP11‐100 M19; Green) of Chromosome 19. (A) Metaphase FISH revealed red and green signals on Chromosome 19. However, the metaphase FISH could not distinguish between Chromosome 19 and inv(19). (B) FISH‐inverted DAPI image of (A). (C) Interphase FISH showed an inverted pattern (Red‐Green/Red‐Green) on inv(19). (D) Schematic representation for the FISH analysis of inv(19). (E–H) FISH analysis of peripheral blood cells using probes of Breakpoint 1 (RP11‐450D10, RP11‐662 N17; Red) and downstream of Breakpoint 2 (RP11‐568 L16; Green) of Chromosome 19. (E) Metaphase FISH revealed red and green signals on Chromosome 19. However, the metaphase FISH could not distinguish between Chromosome 19 and inv(19). (F) Prometaphase FISH with a long chromosome showed an inverted pattern (Red‐Red on Green) on inv(19). (G) Interphase FISH also showed an inverted pattern (Red‐Red‐Green) on inv(19). (H) Schematic representation for the FISH analysis of inv(19).

First, we conducted a TruSight One gene panel (Illumina Inc., San Diego, CA) to identify the cause of MSUD. We identified one heterozygous missense variant (NM_000709.3:c.757G > A, NP_000700.1:p.Ala253Thr) in *BCKDHA* that was inherited from the proband's mother (Figure 1A). However, we could not find any other causative variant in the coding region of *BCKDHA*. Next, total RNA was extracted from the proband's lymphocyte cells and reverse‐transcribed cDNA was used for sequencing analysis. Although no additional variant was identified, the nucleotide found to be heterozygous for c.757G > A in the proband's genome showed only variant A, suggesting that the transcripts were exclusively derived from the maternal allele (Figure 1B). Considering the large indel in the region of *BCKDHA*, we performed copy number analysis using eXome Hidden Markov Model analysis with gene panel data. However, no copy number variants were found in this region. As it was possible that variants in the regulatory region or balanced genomic rearrangement blocked the expression of *BCKDHA*, we performed whole‐genome sequencing. An accumulation of discordant reads was found in Intron 1 of *BCKDHA*, and the paired reads were mapped to 3 Mb downstream of *BCKDHA* (Figure 2A). From the orientation of the paired reads, we expected that the proband would carry a paracentric inversion (Figure 2B). Confirmation by inversion‐specific polymerase chain reaction (PCR) showed that the proband and her healthy father had the same structural rearrangement inv(19)(pter→q13.2::q13.32 → q13.2::q13.32 → qter; Figure 2C). Sanger sequencing of the PCR products indicated that Junction 1 represented a simple blunt end joining, although 4‐bp microhomology (GTGA) was also identified at two nucleotides downstream of the putative breakpoint. In contrast, Junction 2 showed a simple 4‐bp microinsertion (Figure 2D). Based on the sequence information, fluorescence in situ hybridization (FISH) analysis was performed with probes encompassing breakpoints 1 (RP11‐450D10, RP11‐662 N17) and 2 (RP11‐100 M19). As a result, we confirmed inv(19) by observing split signals on the interphase nuclei (Figure 3A–D). In addition, we performed FISH analysis with a probe encompassing breakpoint 1 (RP11‐450D10, RP11‐662 N17) and another located distal to breakpoint 2 (RP11‐568 L16; Figure 3E–H). For the interphase nuclei, the results showed inv(19) with red and green signals in close proximity at Junction 2 (Figure 3G). Although normal metaphase FISH could not distinguish between Chromosomes 19 and inv(19) (Figure 3E), the prometaphase chromosome revealed inv(19), as evidenced by the separated red signals (Figure 3F).

## 2 DISCUSSION

Here, we describe a young female patient with MSUD caused by a compound heterozygous mutation, NM_000709.3:c.757G > A(p.Ala253Thr)/inv(19)(pter→q13.2::q13.32 → q13.2::q13.32 → qter). The c.757G > A (p.Ala253Thr) mutation was previously reported to result in a classic phenotype.^10^ As we did not identify the *BCKDHA* transcripts from the inversion allele, the inversion allele with disruption of Intron 1 possibly produces no protein at all owing to nonsense‐mediated mRNA decay. BCKD consists of three catalytic components, and it is considered that it cannot function well if one of the components changes significantly. Therefore, the present case exhibited a classic phenotype.

The breakpoint junction of the inversion provides mechanistic insight into this genomic rearrangement. The blunt ends at Junction 1 and microinsertion of the unrelated sequence at Junction 2 implicate repair of double‐strand breaks (DSBs) via nonhomologous end joining. However, we identified GTGA microhomology at two nucleotides downstream of the putative breakpoint. Microhomology and microinsertion observed at the junction are the characteristics of the repair mechanism for DSBs or replication errors via alternative nonhomologous end joining; in other words, microhomology‐mediated end joining.^11^ Junction 1 was possibly developed by DSB repair by the usage of 4‐nucleotide microhomology followed by insertion of TG in microhomology‐mediated end joining, or via translesion DNA synthesis at the restart of replication fork collapse with error‐prone DNA polymerase during the repair process.^12^ Notably, small nucleotide mutations were often identified at the junction of the genomic rearrangement with other evidence of replication slippage.^13^ This supports the interpretation of Junction 1, not as blunt‐end ligation, but as utilization of 4 bp microhomology.

In this case, we verified the presence of chromosome inversion by whole‐genome sequencing followed by junction‐specific PCR; however, FISH with probes encompassing the breakpoints was also a powerful tool to visually confirm the inversion. When the breakpoints of inversion are close to each other, confirming the inversion using ordinary metaphase FISH is difficult; thus, interphase FISH is often used for confirmation. However, with interphase FISH, information on chromosomal location is not available, and visually confirmation of the chromosomal structure of the chromosome is sometimes difficult. In this case, when one probe was designed at Breakpoint 1 and the other distal to Breakpoint 2, inversion was confirmed by signals on prometaphase chromosomes that had longer chromosome axes than at metaphase, providing high‐resolution FISH signals.

In this case, gene panel diagnosis revealed that the proband carried a pathogenic missense variant of maternal origin, and the RNA study led us to predict that another mutation of paternal origin was located in the noncoding region of *BCKDHA*. In recent years, low sequencing costs and rapid computing speed have created a standard genetic diagnosis environment for whole‐genome sequencing. Some reports recommend whole‐genome sequencing as the first approach in the diagnosis of unexplained diseases in newborns.^14^ In patients with inborn errors of metabolism, biochemical diagnosis is generally sufficient to treat the clinical symptoms of the patients, but genetic diagnosis is necessary for carrier testing or future prenatal/preimplantation tests. Using the sequence information of the breakpoint junction, we established a junction‐specific PCR that could be used for the detection of this variant in carrier or future prenatal/preimplantation tests. In conclusion, we report the classic form of MSUD with paracentric inversion of Chromosome 19, which disrupts *BCKDHA*.

## AUTHOR CONTRIBUTIONS

**Katsuyuki Yokoi:** retrieved the data and drafted and revised the article. **Yoko Nakajima:** conception and design, analysis and interpretation, and drafting of the article. **Yuta Sudo:** provided chronic phase care for the patient. **Tasuku Mariya:** performed sequence analysis. **Rie Kawamura:** performed cytogenetic analysis. **Makiko Tsutsumi:** performed the eXome Hidden Markov Model. **Hidehito Inagaki:** performed whole‐exome. **Tetsushi Yoshikawa:** provided chronic phase care for the patient. **Tetsuya Ito:** provided neonetal period care for the patient. **Hiroki Kurahashi:** conception and design, analysis, interpretation, and critical revision of the article for important intellectual content.

## CONFLICT OF INTEREST

The author declares that there is no conflict of interest that could be perceived as prejudicing the impartiality of the research reported.

## ETHICS STATMENT

All procedures followed were in accordance with the ethical standards of the responsible committee on human experimentation (institutional and national) and the Helsinki Declaration of 1975, as revised in 2005 (5). The study protocol was approved by the Ethical Review Board for Human Genome Studies of Fujita Health University.

## PATIENT CONSENT

Written informed consent for the publication of medical information and images was obtained from the patient's parents reported in this publication.

## Data Availability

Data and material are available upon request.
